# Anaesthetic Type and Survival in Metastatic Colorectal Cancer: A Nationwide Registry Study

**DOI:** 10.1111/aas.70320

**Published:** 2026-08-23

**Authors:** Ahmed Tarfy, Anna Enlund, Erland Östberg, Hanna Vikman, Anders Berglund, Mats Enlund, Maziar Nikberg

**Affiliations:** ^1^ Department of Surgery and Centre for Clinical Research Västmanland Hospital Västerås Sweden; ^2^ Department of Surgical Sciences Uppsala University Uppsala Sweden; ^3^ Department of Anaesthesia and Intensive Care and Centre for Clinical Research Västmanland Hospital Västerås Sweden; ^4^ Epistat AB Uppsala Sweden; ^5^ Department of Surgical Sciences, Anaesthesiology and Intensive Care Uppsala University Uppsala Sweden; ^6^ ESAIC Onco Anaesthesiology Research Group EuroPeriscope Brussels Belgium

**Keywords:** colorectal neoplasms, propofol, sevoflurane, survival

## Abstract

**Background:**

The impact of anaesthetic choice on survival following metastatic cancer surgery is uncertain. Inhalational anaesthetics, such as sevoflurane, may promote inflammation and tumour progression, while the intravenous agent propofol has been suggested to be anti‐inflammatory. This study aimed to evaluate the association between anaesthetic maintenance with inhalational anaesthesia versus propofol‐based total intravenous anaesthesia and overall survival in patients undergoing surgery for metastatic colorectal cancer.

**Methods:**

Adult patients (≥ 18 years) with metastatic colorectal cancer who underwent elective or emergency surgery under general anaesthesia from 2014 to 2019 were identified in the Swedish Colorectal Cancer Registry and matched with the Swedish Perioperative Registry. Cox regression, propensity score‐matching (2:1, inhalational anaesthesia: propofol), and Kaplan–Meier analysis were used to assess survival.

**Results:**

Of the 1233 included patients, 1011 received maintenance anaesthesia with an inhalational agent, and 222 received propofol. Cox regression analysis identified significant associations between overall survival and advanced age, higher ASA class, emergency surgery, open surgical approach and right‐sided tumour location. Comparison of anaesthetics for the full unmatched cohort observed a statistically significant increased risk of death with propofol (hazard ratio [HR] 1.26, 95% CI 1.02–1.55, *p* = 0.032). Propensity score‐matching yielded 639 matched patients (inhalants 426; propofol 213) with Kaplan–Meier analysis showing comparable survival between groups, with 1‐ and 3‐year survival rates of 75% and 43% for inhalants versus 73% and 41% for propofol. In the matched cohort, the HR for propofol versus inhalants in a Cox regression analysis was 1.13 (95% CI 0.89–1.42, *p* = 0.32).

**Conclusion:**

An apparent association between propofol maintenance and increased mortality observed in the full unmatched cohort was not confirmed in the propensity score‐matched cohort. Poorer survival was independently associated with advanced age, higher ASA class, right‐sided tumours and open surgical approach.

**Editorial Comment:**

It is still not known if clinical anaesthetic exposure can affect cancer outcomes. This retrospective analysis of metastatic colorectal cancer surgical cases looked for associations for anaesthesia management factors, or maintenance of anaesthesia with propofol or inhalational agents, and long‐term survival. When the anaesthesia exposure cases were matched for other known mortality risk factors, there was no anaesthesia‐related mortality risk difference over time between groups in this cohort.

## 1 Introduction

Cancer remains one of the leading causes of death worldwide, with most patients ultimately dying from metastatic disease [1]. Surgery is a cornerstone in the management of solid tumours, with an estimated 60%–80% of cancer patients requiring surgery during the course of their disease [2, 3]. However, both surgical trauma and anaesthetic agents can alter and suppress the immune system, creating a perioperative environment that favours tumour persistence and metastatic spread [4, 5, 6].

The potential influence of anaesthetic technique on cancer outcomes has been debated for more than two decades [7, 8]. Inhalational anaesthetics have been suggested to promote tumour progression by inducing pro‐inflammatory responses, upregulating hypoxia‐inducible factor‐1α and impairing DNA repair [9, 10, 11, 12]. In contrast, propofol has been associated with protective effects, including suppression of pro‐inflammatory cytokines, preservation of natural killer cell function, inhibition of cancer cell motility and attenuation of perioperative immunosuppression [13, 14].

Early retrospective studies indicated that anaesthetic choice might influence long‐term survival [15, 16]. However, recent large randomised clinical trials in patients undergoing breast cancer surgery found no difference in long‐term survival between propofol‐ and sevoflurane‐based anaesthesia [17, 18]. The generalisability of these findings to colorectal cancer may be limited due to differences in tumour biology and anaesthesia duration.

In colorectal cancer, the impact of anaesthetic choice on oncological outcome remains uncertain and although randomised trials are ongoing, their results will take time to become available. Registry‐based and retrospective studies have yielded inconsistent findings and most available evidence concerns non‐metastatic disease [19, 20]. Clinical and experimental observations indicate that surgery may, in some patients, accelerate the progression of metastatic disease, possibly through surgical stress and anaesthetic factors that transiently promote tumour growth [21, 22].

Given the inconsistency of prior findings and the lack of well‐designed large population‐based studies, we aimed to examine whether the choice of anaesthetic technique—inhalational‐based anaesthesia (IA) or propofol based total intravenous anaesthesia (TIVA)—affects survival among patients undergoing surgery for metastatic colorectal cancer. The co‐primary endpoints were 1‐ and 3‐year overall survival, defined as the time from surgery to death from any cause. The secondary endpoint was 90‐day mortality.

## 2 Methods

Following approval of the Swedish Ethical Review Authority (Dnr: 2019‐03728), we combined data from the Swedish Colorectal Cancer Registry and the Swedish Perioperative Registry. The requirement for informed consent was waived due to the retrospective registry‐based design of the study. The Swedish Colorectal Cancer Registry includes detailed information on all patients diagnosed with colorectal cancer in Sweden, such as tumour stage, surgical treatment, oncological therapy, follow‐up and survival, with nationwide coverage above 99% and high validity [23]. The Swedish Perioperative Registry collects data across the entire perioperative pathway, including preoperative assessments, intraoperative variables and anaesthetic technique for maintenance. Coverage during the study period was approximately 99% [24]. Both registries use the unique Swedish personal identification number, enabling accurate linkage and ensuring high data quality. The study was reported in accordance with the STROBE guidelines for observational studies.

We identified all patients aged ≥ 18 years with synchronous metastatic colorectal cancer who underwent resection of the primary tumour between 2014 and 2019. Only patients who received a single anaesthetic maintenance technique were included in the analysis. For patients undergoing more than one eligible procedure during the study period, the earliest was included. All eligible patients during the study period were included, and no formal a priori sample size calculation was performed.

The exposure variable was the anaesthetic maintenance technique: inhalational based or propofol based. Covariates of interest included age, sex, ASA physical status (I–II, III, IV), year of surgery, estimated blood loss, urgency of surgery (elective or emergency), surgical approach (open or minimally invasive surgery) and tumour location. Tumours were categorised as right‐sided colon (caecum, ascending, hepatic flexure and transverse colon), left‐sided colon (splenic flexure, descending and sigmoid colon) or rectum (≤ 15 cm from the anal verge). Patients with non‐resectional procedures (exploratory laparotomy with or without stoma creation) or local/endoscopic resections were excluded.

Descriptive statistics were used to compare baseline characteristics between treatment groups, both in the full unmatched and the propensity score‐matched populations. Categorical variables were presented as frequencies and percentages, while continuous variables were reported as medians with interquartile ranges (IQR).

To reduce confounding, 1:2 nearest‐neighbour propensity score‐matching without replacement was performed. The propensity score was estimated using a logistic regression model, with treatment type as the dependent variable and the following covariates as independent variables: age at diagnosis, sex, year of surgery, ASA class, urgency of surgery, surgical approach and tumour location. Only complete cases for these variables were included in the matching process. Covariate balance before and after matching was assessed using standardised mean differences and visualised with Love plot. A threshold of 0.1 was used to indicate meaningful imbalance (Figure 2).

Overall survival was estimated using the Kaplan–Meier method, with survival probabilities estimated at one and 3 years for both the full unmatched and the matched cohort. Patients alive at the last follow‐up were censored at that time point. Survival distributions between groups were compared using the log‐rank test.

Multivariable Cox proportional hazards regression models were used to assess the association between treatment type and mortality, with results presented as hazard ratios (HRs) and corresponding 95% confidence intervals (CIs). In the full cohort, multivariable Cox regression included covariates such as age, sex, year of surgery, ASA class, urgency of surgery, surgical approach and tumour location. In the propensity score‐matched cohort, Cox regression was used to account for residual imbalance.

To evaluate the secondary endpoint, we conducted an analysis of 90‐day mortality. For this fixed‐time endpoint, we assessed whether patients had at least 90 days of potential follow‐up, defined as either (i) death within 90 days after surgery or (ii) being alive with ≥ 90 days of follow‐up time. All patients in the study fulfilled this requirement; therefore, no individuals were excluded based on follow‐up duration.

In the original cohort, 90‐day mortality was analysed using multivariable logistic regression, with anaesthetic technique as the primary exposure. The model was adjusted for age, sex, year of surgery, ASA class, urgency of surgery, tumour location and surgical approach.

The propensity score‐matched cohort was analysed using conditional logistic regression, stratified by matching subclass. This approach accounts for the matched design by comparing outcomes within matched strata, thereby providing an unbiased estimate of the association between anaesthetic technique and 90‐day mortality.

All statistical analyses were conducted using R version 4.3.3, with a significance level set at *p* < 0.05.

## 3 Results

A total of 1233 patients with Stage IV colorectal cancer were identified between 2014 and 2019. Of these, 1011 (82%) received inhalational anaesthesia and 222 (18%) received propofol. Baseline characteristics were generally similar, including sex distribution and a median age of 70 years in both groups. Propofol use differed across regions and showed an upward trend over time (Table 1). Differences were observed in ASA class, urgency of surgery, surgical approach and tumour location, which were adjusted for in multivariable and matched analyses.

**TABLE 1:** Baseline characteristics of the study population *before* propensity score‐matching.

Characteristic	Inhalational (*n* = 1011)	Propofol (*n* = 222)	Overall (*n* = 1233)	SMD
Age at diagnosis (years)	70 [61–77]	71 [63–76]	70 [62–77]	0.015
Sex				0.057
Female	445 (44)	104 (47)	549 (45)	
Male	566 (56)	118 (53)	684 (56)	
Healthcare region				0.585
North	74 (7)	16 (7)	90 (7)	
Stockholm/Gotland	228 (23)	27 (13)	255 (21)	
South	157 (16)	31 (14)	188 (15)	
Southeast	141 (14)	21 (10)	162 (13)	
Uppsala/Örebro	263 (26)	42 (19)	305 (25)	
West	148 (15)	85 (38)	233 (19)	
Year of surgery				0.330
2014–2015	187 (19)	18 (8)	205 (17)	
2016–2017	355 (35)	76 (34)	431 (35)	
2018–2019	469 (46)	128 (58)	597 (48)	
ASA class				0.200
I–II	561 (56)	134 (60)	695 (56)	
III	401 (40)	70 (31)	471 (38)	
IV	25 (3)	10 (5)	35 (3)	
Missing	24 (2)	8 (4)	32 (3)	
Urgency of surgery				0.056
Emergency	228 (23)	47 (21)	275 (22)	
Elective	782 (77)	175 (79)	957 (78)	
Surgical resection				0.150
Right‐sided	410 (41)	95 (43)	505 (41)	
Left‐sided	338 (33)	83 (37)	421 (34)	
Rectum	263 (26)	44 (20)	307 (25)	
Bleeding (mL)	200 [50–500]	100 [50–300]	150 [50–450]	0.248
Surgical approach				0.198
Laparoscopic	213 (21)	63 (28)	276 (22)	
Open	798 (79)	158 (71)	956 (78)	

*Note:* Data are presented as median [IQR] for continuous variables and number (percentage) for categorical variables.

Abbreviations: ASA = American Society of Anesthesiologists; SMD = standardised mean differences.

**FIGURE 1:**
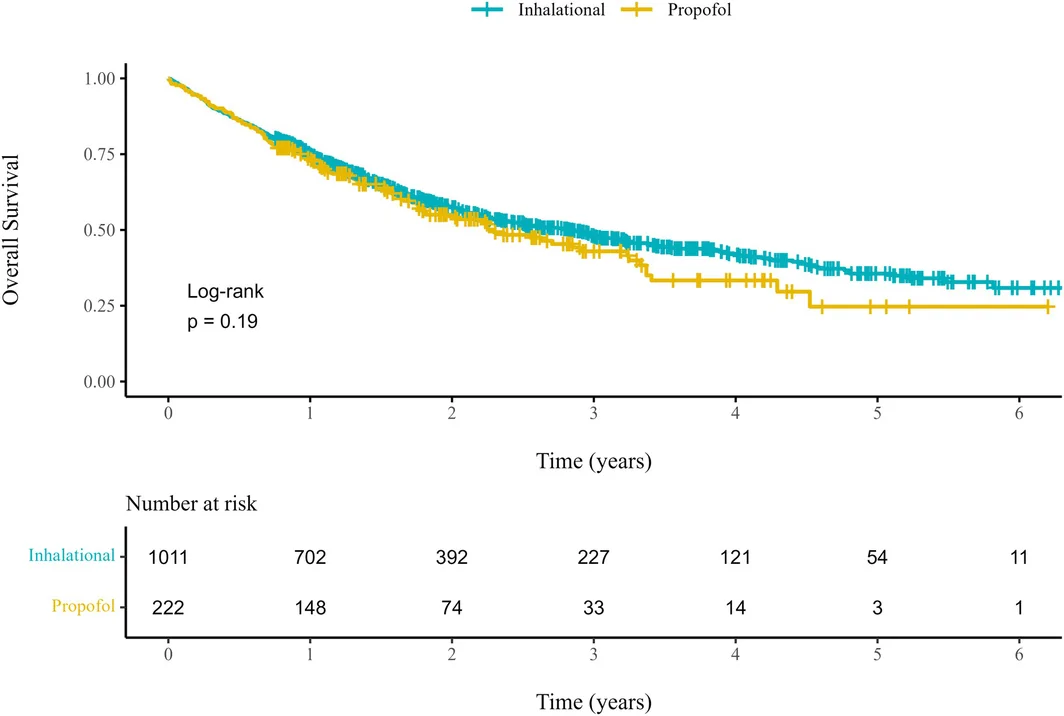
Kaplan–Meier overall survival curves before propensity score‐matching. Survival probabilities at 1 and 3 years were 75% and 48% in Inhalational anaesthesia group and 73% and 43% in the propofol group. No significant difference was observed between groups (log‐rank *p* = 0.19).

Multivariable Cox regression analysis identified independent predictors of increased mortality: advanced age, higher ASA class, emergency surgery, open surgical approach and right‐sided tumour location. In the full unmatched cohort, 1‐ and 3‐year survival was 75% and 48% with inhalational anaesthesia and 73% and 43% with propofol. Kaplan–Meier curves demonstrated overlapping survival distributions with no significant difference between groups (log‐rank *p* = 0.19; Figure 1). In contrast, Cox regression showed that propofol was associated with higher mortality compared with inhalational anaesthesia (HR 1.26, 95% CI 1.02–1.55; *p* = 0.032; Table 2).

**TABLE 2:** Cox regression analysis of mortality in the full unmatched cohort.

Characteristic	HR	95% CI	*p*
Anaesthetic type			
Inhalational	Ref	—	—
Propofol	1.26	1.02–1.55	0.032
Age (per year)	1.02	1.01–1.03	< 0.001
Sex			
Female	Ref	—	—
Male	1.06	0.90–1.25	0.5
Year of surgery			
2014–2015	Ref	—	—
2016–2017	1.06	0.85–1.32	0.6
2018–2019	0.99	0.78–1.26	> 0.9
ASA class			
I–II	Ref	—	—
III	1.26	1.06–1.50	0.010
IV	1.53	0.99–2.36	0.057
Urgency of surgery			
Emergency	Ref	—	—
Elective	0.51	0.42–0.61	< 0.001
Tumour location			
Left colon	Ref	—	—
Right colon	1.56	1.29–1.88	< 0.001
Rectum	0.98	0.77–1.26	> 0.9
Surgical approach			
Laparoscopic	Ref	—	—
Open	1.29	1.02–1.62	0.032

*Note:* Data are presented as hazard ratios (HRs) with 95% confidence intervals (CIs), *n* = 1233.

Abbreviation: ASA = American Society of Anesthesiologists.

**FIGURE 2:**
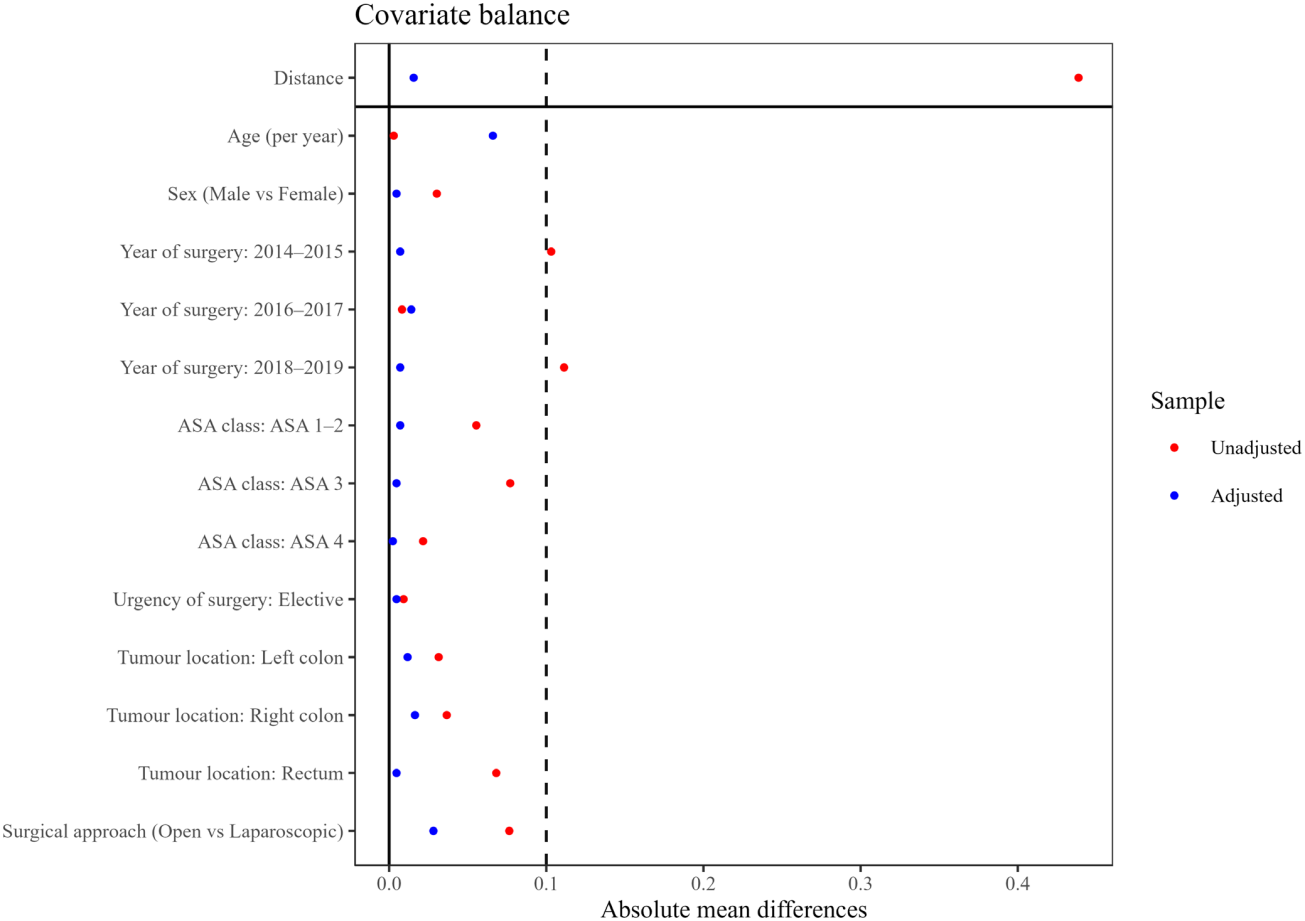
Love plot of standardised mean differences before and after propensity score‐matching. Covariate balance was substantially improved following 1:2 propensity score‐matching. All standardised mean differences were reduced to below 0.1 after matching, indicating good balance between groups.

**FIGURE 3:**
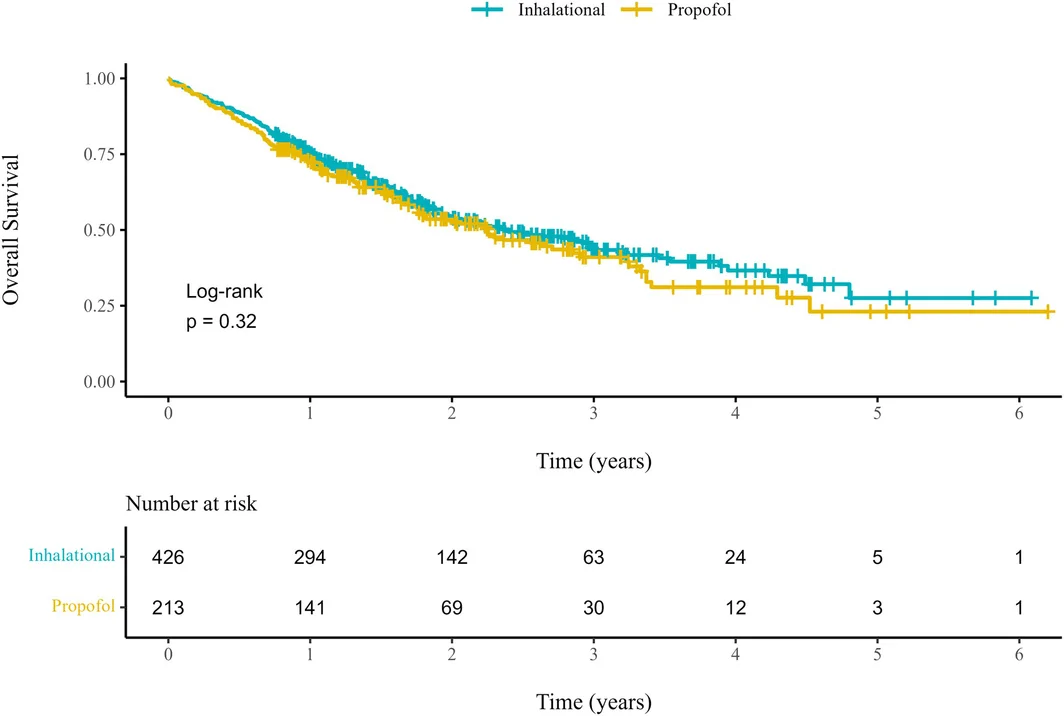
Kaplan–Meier overall survival curves after propensity score‐matching. In the matched cohort (*n* = 639), 1‐ and 3‐year survival rates were 75% and 43% in the inhalational anaesthesia group, compared with 73% and 41% in the propofol group. No significant difference was observed between groups (log‐rank *p* = 0.32).

Propensity score‐matching yielded 639 matched patients (426 inhalants; 213 propofol). Matching achieved good balance across age, sex, ASA class, urgency of surgery, surgical approach and tumour location (Table 3 and Figure 2). The matched cohort analysis revealed 1‐ and 3‐year survival rates of 75% and 43% for inhalants versus 73% and 41% for propofol. Kaplan–Meier curves showed no significant difference (log‐rank *p* = 0.32; Figure 3). Cox regression yielded a HR of 1.13 for propofol versus inhalational agents (95% CI 0.89–1.42, *p* = 0.32).

**TABLE 3:** Baseline characteristics of the study population *after* propensity score‐matching.

Characteristic	Inhalational (*n* = 426)	Propofol (*n* = 213)	Overall (*n* = 639)	SMD
Age at diagnosis (years)	71 [62.25–77.00]	70 [63.00–76.00]	71 [63.00–77.00]	0.068
Sex				0.009
Female	202 (47)	100 (47)	302 (47)	
Male	224 (53)	113 (53)	337 (53)	
Healthcare region				0.577
North	37 (9)	15 (7)	52 (8)	
Stockholm/Gotland	97 (23)	27 (13)	124 (19)	
South	88 (21)	31 (15)	119 (19)	
Southeast	51 (12)	21 (10)	72 (11)	
Uppsala/Örebro	96 (23)	41 (19)	137 (21)	
West	57 (13)	78 (37)	135 (21)	
Year of surgery				0.036
2014–2015	33 (8)	18 (9)	51 (8)	
2016–2017	152 (36)	73 (34)	225 (35)	
2018–2019	241 (57)	122 (57)	363 (57)	
ASA class				0.016
I–II	269 (63)	133 (62)	402 (63)	
III	138 (32)	70 (33)	208 (33)	
IV	19 (5)	10 (5)	29 (5)	
Urgency of surgery				0.011
Emergency	94 (22)	46 (22)	140 (22)	
Elective	332 (78)	167 (78)	499 (78)	
Surgical resection				0.058
Right‐sided	197 (46)	93 (44)	290 (45)	
Left‐sided	147 (35)	79 (37)	226 (35)	
Rectum	82 (19)	41 (19)	123 (19)	
Bleeding (mL)	150 [50–400]	100 [50–300]	150 [50–400]	0.208
Surgical approach				0.063
Laparoscopic	110 (26)	61 (29)	171 (27)	
Open	316 (74)	152 (71)	468 (73)	

*Note:* Data are presented as median [IQR] for continuous variables and number (percentage) for categorical variables.

Abbreviations: ASA = American Society of Anesthesiologists; SMD = standardised mean differences.

For the secondary outcome, the crude 90‐day mortality rate was similar between groups in the full unmatched cohort (6.9% in the inhalational group and 6.8% in the propofol group). In the adjusted logistic regression model, anaesthetic technique was not associated with 90‐day mortality (OR 1.08, 95% CI 0.55–2.00; *p* = 0.80).

In the propensity score‐matched cohort, the crude 90‐day mortality rate was 6.6% in the propofol group versus 5.9% in the inhalational group. The conditional logistic regression showed no significant association between anaesthetic technique and 90‐day mortality (OR 1.14, 95% CI 0.56–2.30; *p* = 0.70).

## 4 Discussion

In this nationwide cohort of patients with metastatic colorectal cancer undergoing surgery, anaesthesia maintenance with propofol was associated with higher mortality compared with inhalational anaesthesia in the multivariable Cox regression analysis of the full unmatched cohort. However, this association was not confirmed in the Cox analysis of the propensity score‐matched group. The observed difference in the full unmatched cohort may reflect baseline imbalances between treatment groups rather than a causal effect of anaesthetic technique. In addition, the lower bound of the relatively wide CI (1.02–1.55) suggests that the observed statistical difference may have been influenced by outcomes in a small number of patients. Consistent with this interpretation, the survival curves demonstrated comparable outcomes between groups after matching. Instead, overall survival was primarily determined by age, ASA class, urgency of surgery, surgical approach and tumour location.

As overall survival in this population appears to be predominantly driven by patient‐ and surgery‐related factors, as outlined above, the influence of anaesthetic choice in metastatic disease is likely small in comparison with these factors. Taken together, the findings from the propensity score‐matched cohort in the present study align with previous reports in non‐metastatic colorectal cancer, in which no cancer‐specific survival advantage has been demonstrated for either anaesthetic technique [20].

Nevertheless, it remains clinically and biologically evident that some patients experience unexpectedly rapid metastatic progression following surgery [21, 22]. This phenomenon may reflect individual variability in immune competence, inflammatory response or tumour biology, potentially rendering certain patients more susceptible to perioperative tumour‐promoting effects.

In clinical practice, the choice of anaesthetic agent is often guided more by individual preference and local institutional routines than by evidence of oncological benefit. This pattern was also reflected in our data, where the use of propofol varied substantially across regions and increased over time. Such variation likely reflects differences in departmental protocols, resource availability and evolving perceptions of propofol as a potentially protective agent, despite the absence of consistent survival benefits demonstrated in clinical studies. Accordingly, year of surgery was included as a covariate to account for temporal changes in clinical practice, including the increasing use of propofol, thereby reducing the risk of potential confounding.

In the broader clinical context, it is important to emphasise that there is currently no strong evidence supporting a survival advantage associated with a transition to propofol‐based anaesthesia in patients with colorectal cancer. Although laboratory and small clinical studies have suggested potential biological benefits of propofol, such as preservation of immune function and attenuation of inflammation [13, 14], these effects have not translated into consistent improvements in long‐term oncological outcomes. The decision to use propofol should therefore be guided by anaesthetic considerations rather than expectations of improved cancer survival. Nevertheless, results from ongoing randomised trials may help clarify whether anaesthetic technique influences survival in patients undergoing surgery for colorectal cancer.

Strengths of this study include its large sample size, nationwide coverage and use of high‐quality registries with validated data. Propensity score‐matching reduced confounding and improved internal validity, making the results more likely to reflect real‐world clinical practice.

Limitations include the retrospective, observational design with potential residual confounding. Perioperative data such as opioid use, transfusions or regional techniques were not assessed. The relatively small number of patients receiving propofol compared with the inhalational group limited the statistical power, particularly in the analysis of the propensity score‐matched group. Furthermore, overall survival rather than cancer‐specific endpoints was used, limiting the ability to assess effects related specifically to cancer progression. Finally, heterogeneity in surgical intent (palliative vs. potential curative intent) complicates interpretation.

In summary, anaesthetic maintenance with propofol was associated with an increased risk of death in the full unmatched cohort; however, this association was not statistically significant in the propensity score‐matched cohort. Survival was independently associated with established prognostic factors, including age, ASA class, urgency of surgery, surgical approach and tumour location, which remained the strongest determinants of outcome in patients with metastatic colorectal cancer.

## Author Contributions

Ahmed Tarfy drafted the manuscript, contributed to the study design and finalised the analyses performed by Hanna Vikman and Anders Berglund. Anna Enlund, Erland Östberg, Mats Enlund and Maziar Nikberg contributed to the study design, revision and supervision. All authors critically reviewed and revised the manuscript and approved the final version.

## Funding

The authors have nothing to report.

## Conflicts of Interest

A.B. has received honoraria for arranging an educational course in collaboration with AstraZeneca. M.E. has received consulting fees from Nimbelle AB and honoraria for lectures from Mälardalen University, serves on the steering committee of the ENCORE trial, and is the initiator and principal investigator of the CAN study. M.N. serves as a colorectal proctor for Intuitive Surgical. The other authors declare no conflicts of interest.

## Data Availability

Anonymised individual participant data underlying the findings of this study may be made available upon reasonable request to the corresponding author, subject to applicable Swedish and EU data protection legislation (GDPR), approval of a sound research proposal, and appropriate ethical approval. Access will require a signed data access agreement outlining conditions for data release, data handling, storage, archiving, publication, and co‐authorship. The study protocol and statistical analysis plan may also be made available upon reasonable request. Data sharing will be considered from 6 months after publication of the present manuscript and for up to 3 years thereafter. Statistical code can be made available upon request.
